# Hypoxia-induced shift in the phenotype of proteasome from 26S toward immunoproteasome triggers loss of immunoprivilege of mesenchymal stem cells

**DOI:** 10.1038/s41419-020-2634-6

**Published:** 2020-06-04

**Authors:** Ejlal Abu-El-Rub, Niketa Sareen, Weiang Yan, Keshav Narayan Alagarsamy, Alireza Rafieerad, Abhay Srivastava, Vincenzo Desiderio, Sanjiv Dhingra

**Affiliations:** 1 Institute of Cardiovascular Sciences, St. Boniface General Hospital Research Centre, Regenerative Medicine Program, Department of Physiology and Pathophysiology, Rady Faculty of Health Sciences, University of Manitoba, Winnipeg, MB R2H2A6 Canada; 2 Department of Experimental Medicine, University of Campania Luigi Vanvitelli, 80138 Naples, Italy

**Keywords:** Cell death, Immunology

## Abstract

Allogeneic mesenchymal stem cells (MSCs) are immunoprivileged and are being investigated in phase I and phase II clinical trials to treat different degenerative and autoimmune diseases. In spite of encouraging outcome of initial trials, the long-term poor survival of transplanted cells in the host tissue has declined the overall enthusiasm. Recent analyses of allogeneic MSCs based studies confirm that after transplantation in the hypoxic or ischemic microenvironment of diseased tissues, MSCs become immunogenic and are rejected by recipient immune system. The immunoprivilege of MSCs is preserved by absence or negligible expression of cell surface antigen, human leukocyte antigen (HLA)-DRα. We found that in normoxic MSCs, 26S proteasome degrades HLA-DRα and maintains immunoprivilege of MSCs. The exposure to hypoxia leads to inactivation of 26S proteasome and formation of immunoproteasome in MSCs, which is associated with upregulation and activation of HLA-DRα, and as a result, MSCs become immunogenic. Furthermore, inhibition of immunoproteasome formation in hypoxic MSCs preserves the immunoprivilege. Therefore, hypoxia-induced shift in the phenotype of proteasome from 26S toward immunoproteasome triggers loss of immunoprivilege of allogeneic MSCs. The outcome of the current study may provide molecular targets to plan interventions to preserve immunoprivilege of allogeneic MSCs in the hypoxic or ischemic environment.

## Introduction

Bone marrow-derived allogeneic (donor-derived) mesenchymal stem cells (MSCs) are considered to be prominent cell type for degenerative diseases and autoimmune disorders^1–5^. MSCs are reported to be immunoprivileged, that allowed transplantation of allogeneic MSCs without the risk of being rejected by host immune system^1,6–11^. These properties of MSCs promoted the concepts of universal young and healthy donor-derived “off-the-shelf” allogeneic cell-based products for older and debilitated patients^12,13^. Infact, in the last 10–15 years several clinical trials have tested the safety and efficacy of allogeneic MSCs based products in phase I and II clinical trials^14–19^. The outcome of most of these trials confirmed safety of transplanted cells^20–22^. However, the long-term follow-ups of many of these clinical trials revealed that allogeneic MSCs were able to exert beneficial effects in the transplanted areas for a short period of time, ultimately the benefits were lost^19,23,24^. One of the major limitations of allogeneic MSCs based therapies is poor survival of transplanted cells in the host tissue^25–28^. Furthermore, the outcome of several studies now confirms that allogeneic MSCs after transplantation in stressful micro-environment of the host tissue, become immunogenic and are rejected by the host immune system that results in poor survival of transplanted cells^28–32^. Therefore, in order to maintain therapeutic benefits of allogeneic MSCs, there is a need to preserve immunoprivilege of transplanted cells in the host tissue.

The immunoprivilege of MSCs is preserved by absence or negligible expression of immune antigen-human leukocyte antigen (HLA)-DR^9,10,31,33^. The HLA-DR molecules are cell surface immune antigens that alert the host immune system to initiate an immune response against transplanted cells or tissues. HLA-DR plays a critical role in T-cell-dependent allo-immune responses by presenting the processed exogenous antigens to T helper (Th) cells^31,34,35^. Therefore, HLA-DR has been implicated as the major contributing factor in allograft rejection. Although HLA-DR is expressed constitutively on antigen-presenting cells (monocytes/macrophages, B cells, and dendritic cells), this molecule can be induced in most cell types and tissues in the presence of pro-inflammatory cytokines e.g. IFN-γ or under stressful conditions^31,36–38^. We recently reported in rat and human MSCs that exposure to hypoxia or ischemic conditions was associated with upregulation of HLA-DRα or MHC-II and loss of immunoprivilege of allogeneic MSCs^31^. Hypoxia or ischemic environment is a common underlying condition of many diseased or injured tissues. In this study, we examined the mechanisms of hypoxia-induced upregulation and activation of HLA-DRα in allogeneic human MSCs. We report for the first time that exposure to hypoxic environment led to formation of immunoproteasome in MSCs which is responsible for activation of HLA-DRα and loss of immunoprivilege of allogeneic MSCs.

## Results

### Hypoxia causes downregulation of 19S regulatory subunits and 20S proteolytic core subunits of 26S proteasome

**Fig. 1:**
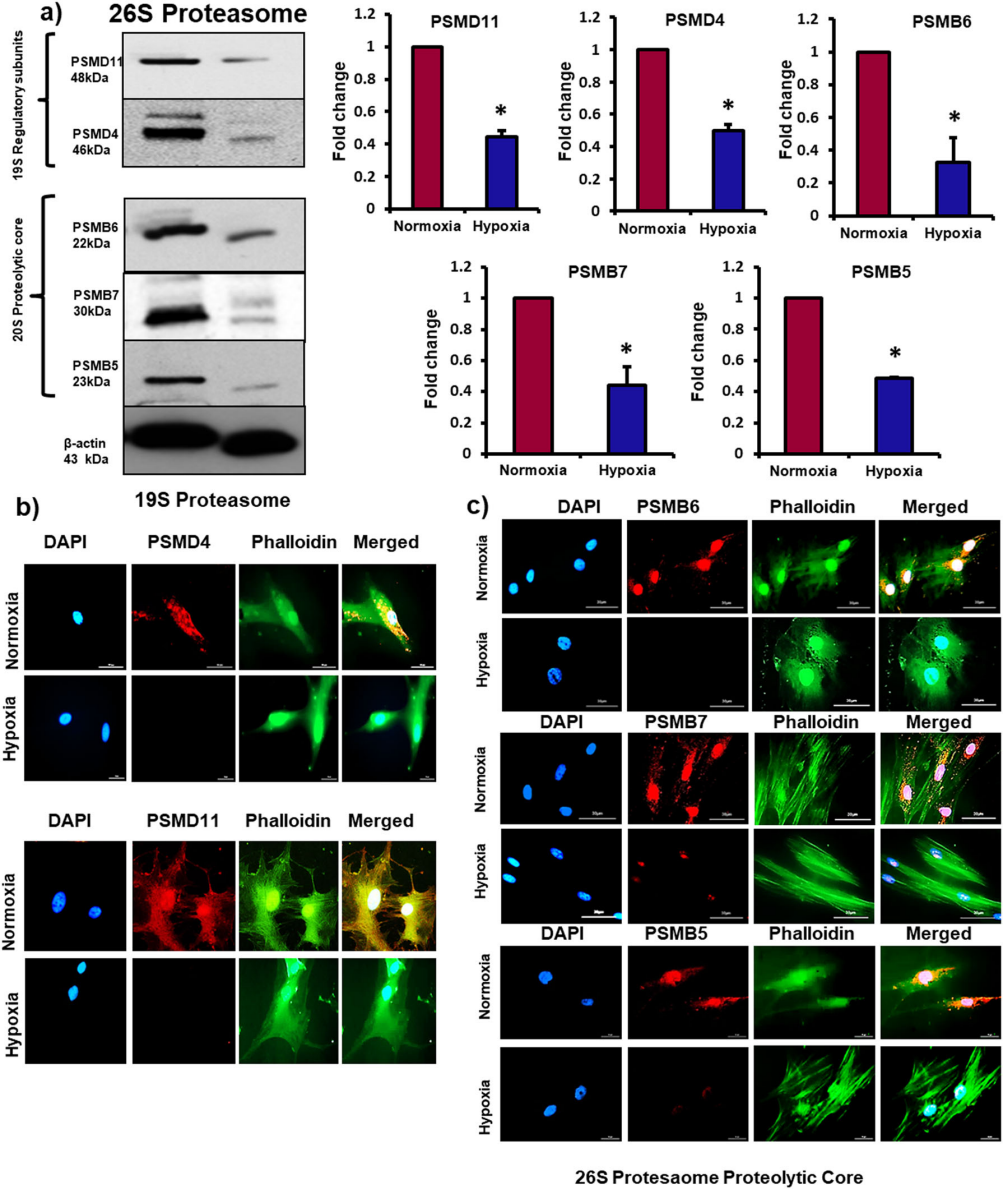
Exposure to hypoxia causes downregulation of regulatory subunits and proteolytic core subunits of 26S proteasome in MSCs. Human bone marrow-derived MSCs were incubated in hypoxia chamber for 24 h. **a** Protein levels of PSMD11, PSMD4 (Rpn10), PSMB6 (β1), PSMB7 (β2), and PSMB5 (β5) as measured by Western blot showed a significant decrease in hypoxic MSCs compared to normoxic cells; *n* = 3. **b**, **c** Immunofluorescence images exhibited a significant decrease in the expression of PSMD4 (Rpn10), PSMD11, PSMB6 (β1), PSMB7 (β2) and PSMB5 (β5) in hypoxic MSCs compared to normoxic cells; *n* = 4. *p < 0.05 compared to normoxic MSCs. Each experiment was repeated 3–4 times.

**Fig. 2:**
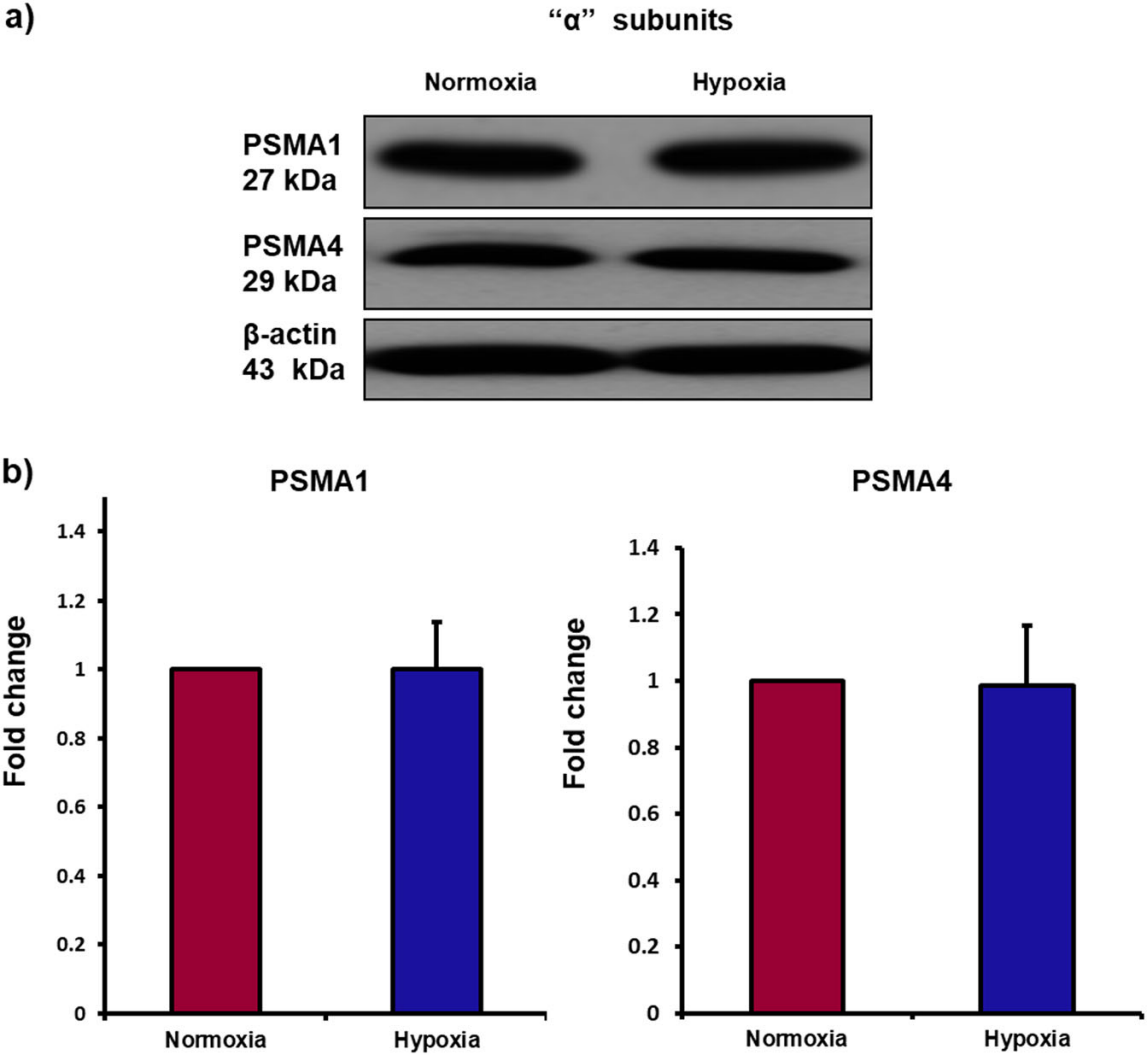
Expression of “α” subunits of 26S proteasome did not change in MSCs after exposure to hypoxia. Human bone marrow-derived MSCs were incubated in a hypoxia chamber for 24 h. The protein levels of 26S proteasome “α” subunits, PSMA1 (α6) and PSMA4 (α3) were detected by Western blot. **a**, **b** Protein levels of PSMA1 (α6) and PSMA4 (α3) did not change in normoxic vs. hypoxic MSCs; *n* = 3. Each experiment was repeated 3 times.

We recently reported in human MSCs that 26S proteasome-mediated degradation of HLA-DRα maintains absence or low levels of this molecule on MSCs surface and preserves immunoprivilege of allogeneic MSCs^31^. Exposure to hypoxic environment was responsible for upregulation of HLA-DRα and immunogenicity of MSCs. These exciting findings prompted us to investigate the fate of 26S proteasome in MSCs under hypoxic conditions and its effects on immunoprivilege of MSCs. The 26S proteasome is composed of a regulatory unit 19S and proteolytic core containing 20S. The 19S regulatory unit receives ubiquitinated target protein and transfers it to the proteolytic core of 20S where the target protein is processed and degraded^39,40^. The deubiquitination proteins PSMD11 and PSMD4 (or Rpn10), which are present in 19S unit, play an important role in processing of target protein^41,42^. In the current study, we found a significant decrease in the expression of these two subunits (Fig. 1a, b). The 20S particle contains three subunits β1 (or PSMB6), β2 (or PSMB7) and β5 (or PSMB5) which are responsible for mediating proteolytic activities of 20S^39,43^. We found a significant decrease in the expression of these three proteins (Fig. 1a, c). These findings demonstrate that exposure to hypoxia leads to downregulation of proteasome regulatory subunits and proteolytic subunits. Interestingly, the “α” subunits which form outer ring of 20S proteasome-α3 (PSMA4) and α6 (or PSMA1) did not change significantly in hypoxic hMSCs compared to normoxic cells (Fig. 2a, b). The α subunits interact with regulatory subunits to mediate proteolytic activities of the proteasome.

### Exposure to hypoxia causes formation of immunoproteasome in MSCs

**Fig. 3:**
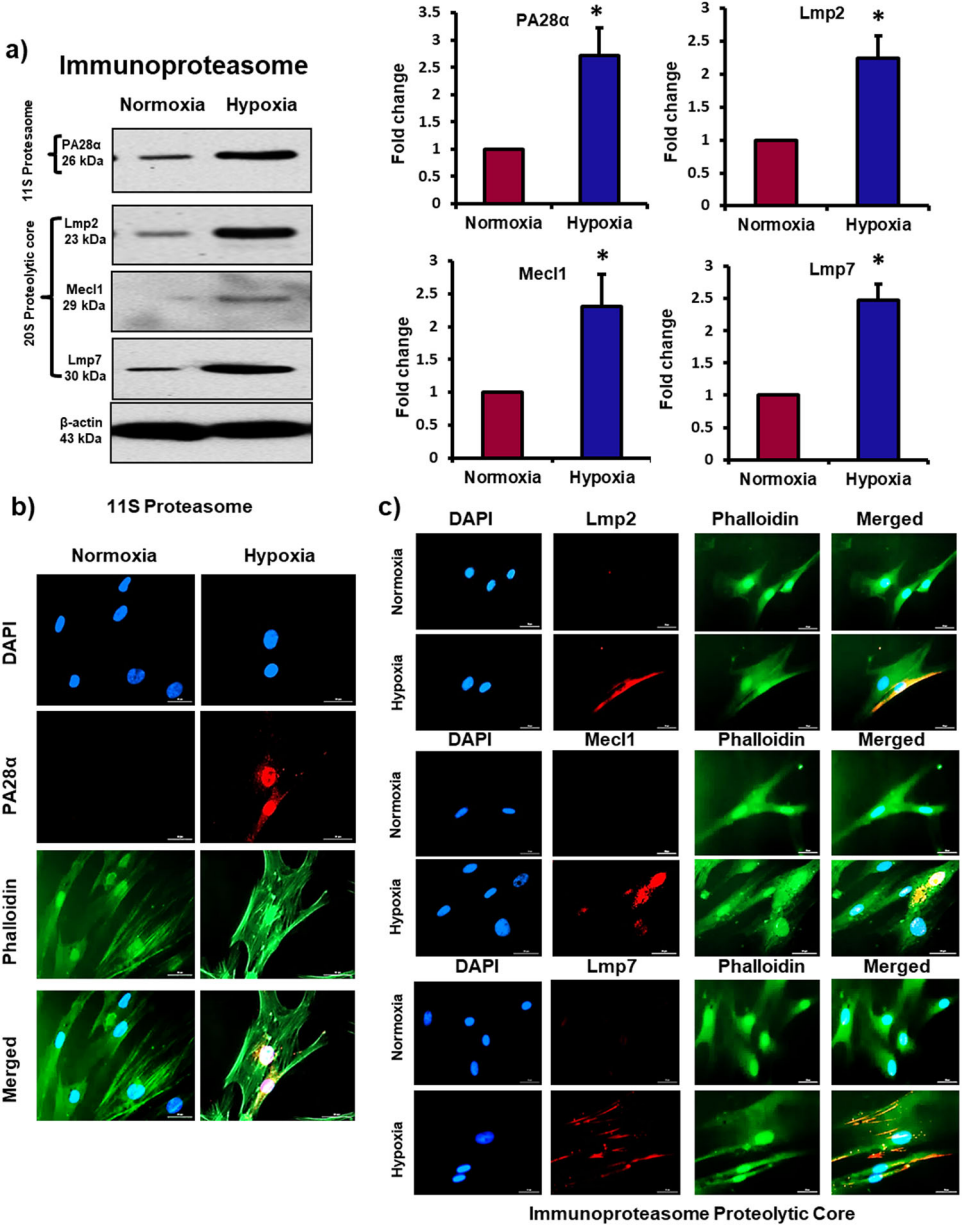
Exposure to hypoxia causes formation of immunoproteasome in MSCs. Human bone marrow-derived MSCs were incubated in a hypoxia chamber for 24 h. **a** Western Blot analysis of PA28α (11S), LMP2 (β1i), MECL1 (β2i), and LMP7 (β5i) showed a significant increase in protein levels in hypoxic MSCs compared to normoxic cells; *n* = 3. **b**, **c** Immunofluorescence images displayed a significant increase in the expression of PA28α (11S), LMP2 (β1i), MECL1 (β2i), and LMP7 (β5i) in hypoxic MSCs compared to normoxic cells; *n* = 4. *p < 0.05 compared to normoxic MSCs. Each experiment was repeated 3–4 times.

Interestingly, we found that in addition to downregulation 26S subunits, exposure to hypoxia in MSCs was associated with the upregulation of 11S particle (or proteasome activator 28α-PA28α). 11S is a regulatory subunit which is reported to replace 19S and bind to 20S particle to form an alternative proteasome “immunoproteasome”^44^. The immunoproteasome is an inducible form of proteasome which is derived from the constitutive 26S proteasome under stress conditions^45,46^. The formation of functional proteasome is a complex process it involves multiple subunits to bind together to form an active complex. Furthermore, the process of switch in the 26S proteasome toward immunoproteasome is quite dynamic, β1 is replaced with iβ1 (large multifunctional peptidase 2, LMP2 or PSMB9), β2 is replaced with iβ2 (multi-catalytic endopeptidase complex-like-1, MECL-1, or PSMB10), and β5 is replaced with iβ5 (large multifunctional peptidase 7, LMP7 or PSMB8), which collectively form the proteolytic core of immunoproteasome^47,48^. In the current study, we measured the expression of LMP2, MECL1 and LMP7 in MSCs after exposure to hypoxia. There was a dramatic increase in the level of these proteins in hypoxic MSCs compared to normoxic cells (Fig. 3a–c).

**Fig. 4:**
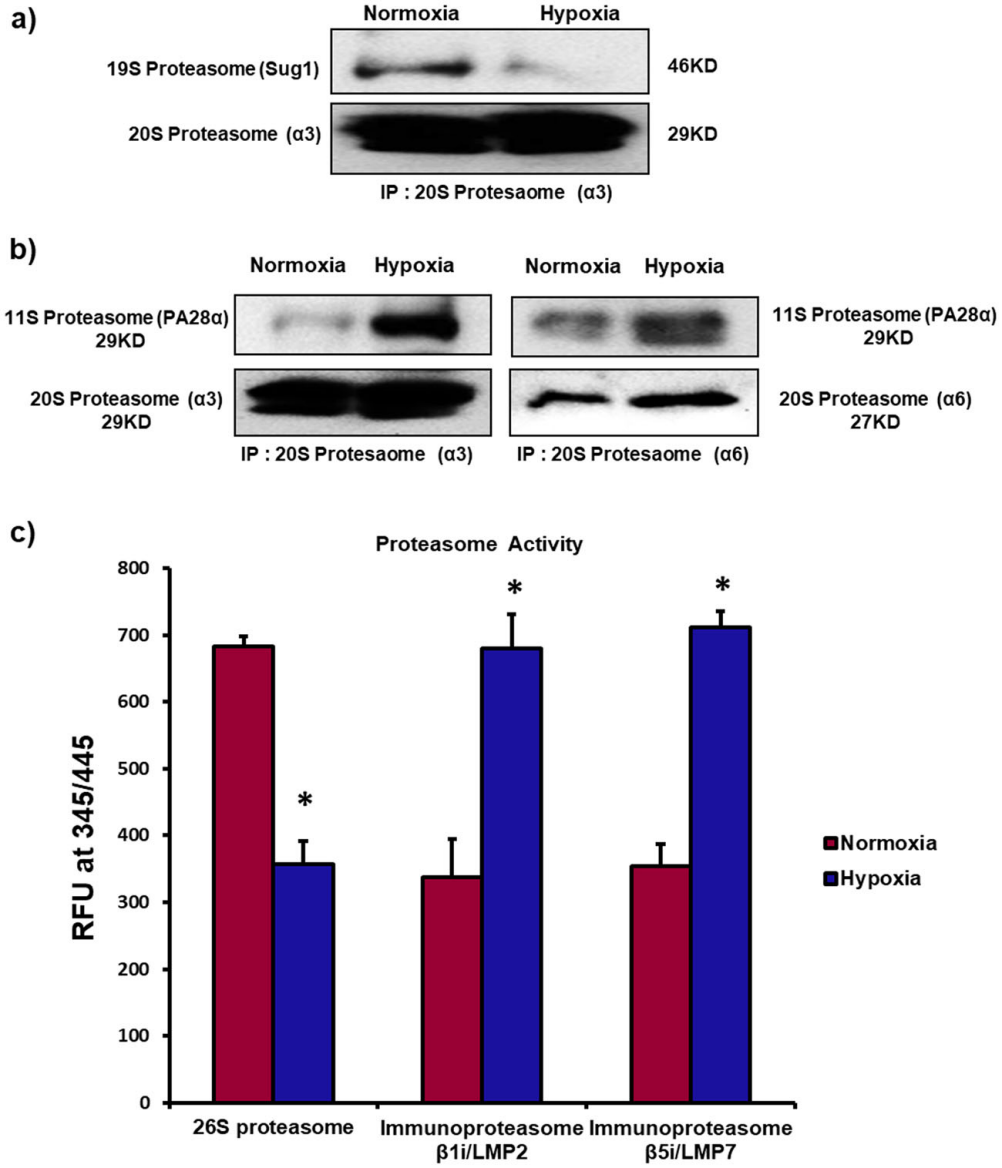
Exposure to hypoxia causes conversion of 26S proteasome to immunoproteasome in MSCs. Human bone marrow-derived MSCs were incubated in a hypoxia chamber for 24 h. **a**, **b** Co-immunoprecipitation analysis was performed in cell lysates to study binding between 19S and 20S subunits; as well as binding between 11S and 20S subunits. **a** The binding affinity between 19S proteasome (Sug1) and 20S proteasome α3 (PSMA4) decreased significantly in hypoxic MSCs compared to normoxic cells. **b** The binding between 11S subunit (PA28α) and α3 (PSMA4); as well as 11S subunit (PA28α) and α6 (PSMA1) increased in hypoxic MSCs vs. normoxic MSCs. **c** Proteasome degradation activities of 26S proteasome and immunoproteasome were measured using specific substrates for these two proteasomes: SUC-LLVY-AMC (specific for 26S proteolytic function), and Ac-PAL-AM as well as Ac-ANW-AMC (specific substrates for proteolytic activities of immunoproteasome subunits β1i/LMP2 and β5i/LMP7). There was significant decrease in 26S proteasome activity in hypoxic MSCs compared to normoxic MSCs. However, immunoproteasome activity significantly increased in hypoxic MSCs compared to normoxic cells; *n* = 5. *p < 0.05 compared to normoxic MSC. Each experiment was repeated 3–4 times.

To further verify that exposure to hypoxia in MSCs lead to formation of immunoproteasome, we performed co-immunoprecipitation (IP) analysis to determine binding between 19S regulatory particle (Sug1 or PSMC5) and the 20S “α” subunits or PSMA4. Our data displayed a notable downregulation in the binding between Sug1 and α3 in hypoxic MSCs in comparison to the normoxic MSCs (Fig. 4a). On the other hand, the co-IP binding assay between 11S regulatory particle (PA28α) and 20S “α” subunits (both α3 and α6) elucidated a remarkable increase in binding between 11S proteasome and α3 (PSMA4) as well as α6 (PSMA1) in hypoxic MSCs compared to normoxic cells (Fig. 4b). These data confirm that in human MSC exposure to hypoxia lead to disassembly of 26S proteasome and formation of immunoproteasome.

Furthermore, in order to validate these data, we analyzed degradation activities of 26S proteasome and immunoproteasome using specific substrates for these two proteasomes: SUC-LLVY-AMC (specific substrate for 26S proteolytic function), and Ac-PAL-AM as well as Ac-ANW-AMC (specific substrates for proteolytic activities of immunoproteasome subunits β1i/LMP2 and β5i/LMP7)^49,50^. The degradation activity of 26S proteasome decreased significantly in hypoxic MSCs compared to normoxic cells (Fig. 4c). However, the proteolytic activity of immunoproteasome in terms of β1i/LMP2 and β5i/LMP7 activities remarkably elevated in hypoxic MSCs in comparison to normoxic cells (Fig. 4c). These proof of concept data further validated hypoxia induced switch in the phenotype of proteasome degradation machinery from 26S proteasome to immunoproteasome.

### Hypoxia-induced switch in the phenotype of proteasome from 26S to immunoproteasome leads to upregulation and activation of HLA-DRα in MSCs

**Fig. 5:**
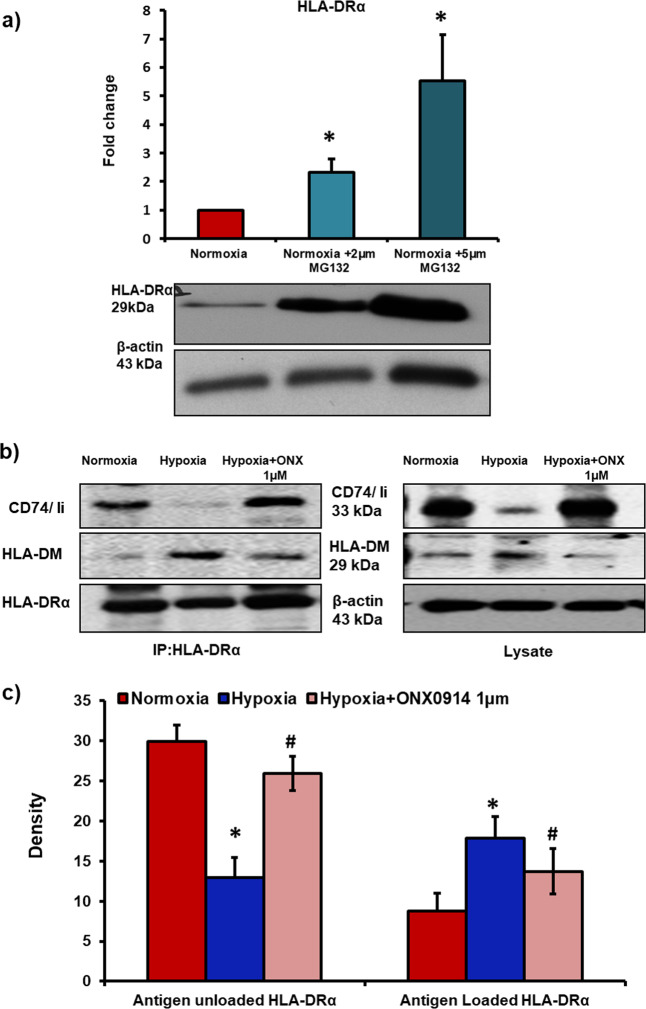
Hypoxia-induced switch in the phenotype of proteasome from 26S to immunoproteasome leads to upregulation and activation of HLA-DRα in MSCs. **a** Human bone marrow-derived MSCs were treated with 26S proteasome inhibitor (MG132, 2 μM and 5 μM) for 24 h. Western blot analysis revealed a significant increase in HLA-DRα protein levels in 26S proteasome inhibited normoxic MSCs; *n* = 3. **b**, **c** MSCs were incubated in hypoxia chamber with or without immunoproteasome inhibitor (Onx0914 1 µM for 4 h). Next, co-immunoprecipitation assay was performed in cell lysates to measure- the binding between HLA-DRα and invariant chain- Ii/CD74 (to measure the levels of immature HLA-DRα, or antigen unloaded HLA-DRα); and the binding between HLA-DRα and HLA-DM (to measure levels of activated HLA-DRα, or antigen loaded HLA-DRα). Inhibition of immunoproteasome increased the binding between HLA-DRα and Ii/CD74; and decreased the binding between HLA-DRα and HLA-DM in hypoxic MSCs; *n* = 3. *p < 0.05 compared to normoxic MSCs, ^#^*p* < 0.05 compared to hypoxic MSCs. Each experiment was repeated 3–4 times.

In the next experiments, we wanted to investigate the effect of hypoxia-induced change in the phenotype of proteasome from 26S to immunoproteasome on HLA-DRα expression in MSCs. Therefore, we blocked 26S and immunoproteasome in MSCs and assessed the expression of HLA-DRα. The inactivation of 26S proteasome using a pharmacological inhibitor (MG132, 2 μM and 5 μM for 24 h) upregulated HLA-DRα expression in normoxic MSCs (Fig. 5a). Previously, immunoproteasome is reported to play a crucial role in MHC-I antigen presentation by degrading immunogenic peptides which help in loading of MHC-I. However, the role of immunoproteasome in HLA-DRα or MHC-II regulation and activation in MSCs has not been investigated yet. To explore whether immunoproteasome play a role in regulating HLA-DRα, we measured HLA-DRα protein levels in hMSCs before and after blocking immunoproteasome. We employed two independent methods to block immunoproteasome in hMSCs, using pharmacological inhibitor (ONX0914, 0.5 μM and 1 μM for 4 h) and siRNA mediated knock down of LMP2 and LMP7 genes. Both LMP2 and LMP7 subunits are crucial for proteolytic activity of immunoproteasome, and siRNA mediated knock down of both these subunits prevented hypoxia-induced increase in immunoproteasome activity (Supplementary Fig. 1a, b). Our data revealed a marked increase in HLA-DRα protein levels in hypoxic hMSCs compared to normoxic cells, and pharmacological as well as siRNA mediated inhibition of immunoproteasome significantly downregulated HLA-DRα expression in hypoxic hMSCs (Supplementary Figs. 1b and 2a, b). These findings confirm that hypoxia-induced switch in the phenotype of 26S proteasome toward immunoproteasome upregulates HLA-DRα in hMSCs.

HLA-DRα activation requires its conversion from immature state to mature antigen. Immature HLA-DRα binds to invariant chain- Ii/CD74, that masks the antigen-binding groove, and antigenic peptides cannot bind to HLA-DRα^51,52^. On the other hand, for maturation and activation of HLA-DRα, the Ii/CD74 is replaced with antigenic peptide HLA-DM^53^. To explore the role of immunoproteasome in HLA-DRα activation in hypoxic MSCs, we performed co-immunoprecipitation assay. Our data revealed that the binding between HLA-DRα and Ii/CD74 in hypoxic MSCs increased in the presence of immunoproteasome inhibitor (Fig. 5b, c), on the other hand the binding between HLA-DRα and HLA-DM decreased in hypoxic MSCs when immunoproteasome activity was blocked (Fig. 5b, c). Therefore, hypoxia-induced formation of immunoproteasome is responsible for maturation and activation of HLA-DRα in MSCs.

### Hypoxia-induced formation of immunoproteasome is responsible for loss of immunoprivilege of MSCs

**Fig. 6:**
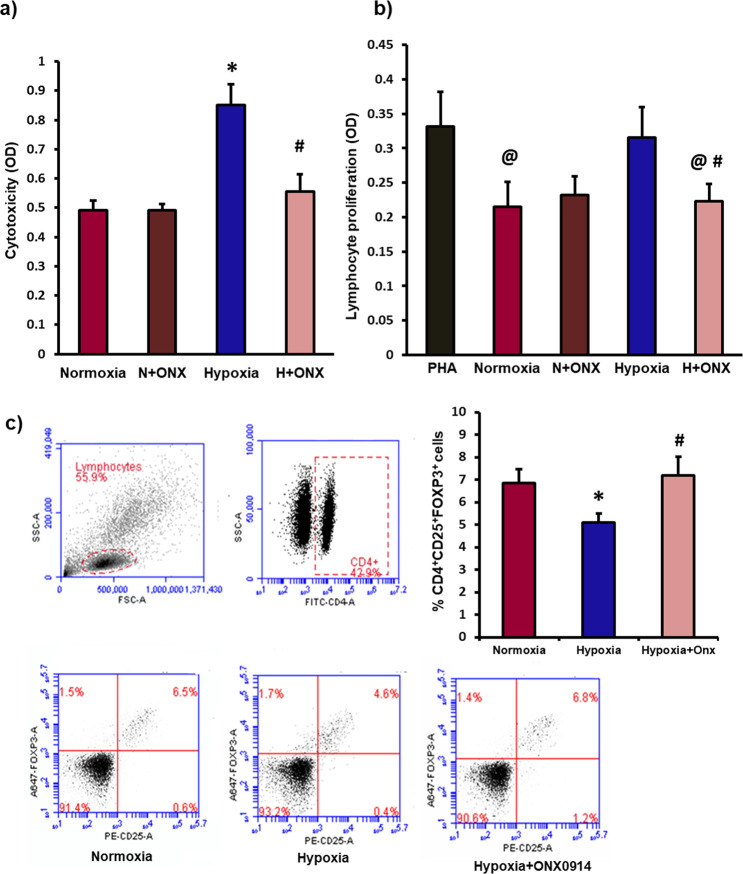
Hypoxia-induced formation of immunoproteasome is responsible for loss of immunoprivilege of MSCs. To determine the immunogenicity of MSCs, normoxic and hypoxic human MSCs (with or without immunoproteasome inhibitor- Onx0914 1 µM for 4 h) were co-cultured with allogeneic leukocytes at a ratio 1:10 for 72 h. **a** Leukocyte mediated cytotoxicity in MSCs (LDH release) increased significantly in hypoxic MSCs vs. normoxic cells, which was rescued by inhibition of immunoproteasome. *n* = 5. **b** The effect of MSCs on leukocyte proliferation was measured using WST1 proliferation assay kit. After 72 h of co-culture, normoxic MSCs were able to decrease leukocyte proliferation compared to control (PHA treated leukocytes). However, hypoxia treated MSCs had no effect on leukocyte proliferation, immunoproteasome inhibited hypoxic MSCs significantly decreased leukocytes proliferation. *n* = 10. **c** After 72 h of co-culture, the effect of MSCs on CD4^+^CD25^+^FOXP3^+^ Treg cell induction in a mixed leukocyte population was assessed by flow cytometry. The number of Treg cells decreased after co-culture with hypoxic MSCs. However, co-culture with immunoproteasome inhibited hypoxic MSCs increased the number of Treg cells. *n* = 3. *p < 0.05 compared to normoxic MSC; ^@^*p* < 0.05 compared to PHA group; ^#^p < 0.05 compared to hypoxic MSCs, each experiment was repeated 3–4 times.

In order to explore the effect of hypoxia-induced formation of immunoproteasome on immunoprivilege of MSCs, the cells were treated with immunoproteasome inhibitor (Onx0914, 1 µm for 4 h) or transfected with siRNA to block LMP2 and LMP7 subunits of immunoproteasome, and immunogenicity of MSCs was studied under normoxic and hypoxic conditions. To assess immunogenicity, hMSCs were exposed to hypoxia and then co-cultured with allogenic leukocytes for 72 h and leukocyte mediated cytotoxicity in MSCs was evaluated by measuring the amount of lactate dehydrogenase released and Annexin V apoptosis assay. The level of cytotoxicity increased significantly in hypoxic MSCs compared to normoxic cells (Fig. 6a, Supplementary Fig. 3a). However, pharmacological or siRNA mediated inhibition of immunoproteasome activity prevented hypoxia-induced increase in leukocyte mediated cytotoxicity in hMSCs (Fig. 6a, Supplementary Fig. 3a). Interestingly, the presence of immunoproteasome inhibitor had no effect on the level of cytotoxicity in normoxic MSC (Fig. 6a). This observation further verified the formation of immunoproteasome in hypoxic MSCs. Bone marrow MSCs have the ability to downregulate leukocyte proliferation and suppress allo- immune responses^10,33^. In the current study, we found that after 72 h of co-culture with leukocytes, normoxic MSCs were able to suppress leukocyte proliferation (Fig. 6b). Hypoxia exposed MSCs were unable to suppress leukocyte proliferation (Fig. 6b). However, immunoproteasome inhibited hypoxic MSCs were able to suppress leukocyte proliferation (Fig. 6b, Supplementary Fig. 3b).

MSCs are also reported to suppress allo-immune responses by promoting phenotype change from cytotoxic T cells toward immunosuppressive regulatory T (Treg) cells^54,55^. Treg cells are known to suppress the proliferation of cytotoxic T cells and promote immune tolerance. We counted the number of CD4^+^CD25^+^FOXP3^+^ Treg cells in a mixed leukocyte population after co-culture with allogeneic MSCs by flow cytometry. The Treg cell number decreased after co-culture with hypoxia exposed MSCs compared to normoxic MSCs (Fig. 6c). However, immunoproteasome inhibited hypoxic MSCs were able to upregulate Treg cell number in a mixed leukocyte population (Fig. 6c). Therefore, hypoxia-induced formation of immunoproteasome leads to upregulation and activation of HLA-DRα and loss of immunoprivilege of MSCs.

## Discussion

Bone marrow-derived allogeneic MSCs are being investigated in phase I and phase II clinical trials for several degenerative diseases^3,18,56^. Despite encouraging therapeutic benefits after MSCs transplantation in patients, overall excitement of MSCs based therapies has lately come down. Major hurdle in bringing allogeneic MSCs to the clinic is poor survival of cells after transplantation in the host tissue^24,28^. MSCs were considered to be favorite cell type to treat degenerative diseases because these cells were initially reported to be immunoprivileged^9,33^. Therefore, the universal belief was that allogeneic transplants would be possible without MSCs being rejected by host immune system. However, the outcome of recent allogeneic MSCs based preclinical studies and clinical trials suggested that cells after transplantation in the host microenvironment become immunogenic and are rejected by host immune system^30,31^. Immunoprivilege of MSCs is preserved by absence or negligible expression of cell surface immune antigen HLA-DRα, that allows escape of transplanted MSCs from host immune system. HLA-DRα is responsible for antigen presentation which is a critical step to alert host CD4^+^ T-cells to initiate allo-immune response against allograft. We have previously reported that 26S proteasome mediated degradation of HLA-DRα maintains absence of this molecule on MSC surface and preserves immunoprivilege of cells^31^. We have also reported that exposure to hypoxic or ischemic environment is responsible for loss of immunoprivilege^31^. Hypoxic or ischemic injury is related to several pathological conditions at organ or tissue level such as myocardial infarction, stroke and peripheral vascular disease. Therefore, effect of hypoxic or ischemic environment on immunological behavior of MSCs needs to be studied in detail to preserve immunoprivilege of transplanted cells under pathological conditions. In the current study, we investigated the effect of hypoxic environment on proteasome complex and immunoprivilege of allogeneic MSCs. The 26S proteasome is a master degradation system inside a cell, it contains 19S regulatory particle and 20S core particle. The 26S proteasome activity requires coordinated action of 19S and 20S assembly for carrying out degradation and proteolysis of ubiquitinated proteins. Basically, 19S particle receives ubiquitinated target protein and PSMD11 and PSMD4 subunits of 19S regulatory unit deubiquitinate the target protein and transfer it to the proteolytic core of 20S where the target protein is processed and degraded^39,43^. The 20S particle contains three subunits β1 (or PSMB6), β2 (or PSMB7) and β5 (or PSMB5) which are responsible for proteolytic activities of 26S proteasome^45,48^. In the current study, there was a significant decrease in the expression of PSMD11, PSMD4, and proteolytic subunits β1, β2 and β5. Therefore, exposure to hypoxic environment leads to dysfunction of 26S proteasome degradation machinery in allogeneic MSCs.

Furthermore, in the current study, we found that hypoxic stress leads to formation of immunoproteasome in MSCs. Immunoproteasome is an inducible form of proteasome which is expressed in immune cells under stress conditions or after exposure to pro-inflammatory cytokines^44,46^. Current study is the first to report formation of immunoproteasome in MSCs under hypoxic conditions. Interestingly, we found that blocking immunoproteasome formation prevented hypoxia-induced increase in immunogenicity of allogeneic MSCs. Therefore, hypoxia-induced inactivation of 26S proteasome and upregulation of HLA-DRα is not sufficient to induce immunogenicity of MSCs, instead formation of immunoproteasome in response to treatment with hypoxia is a crucial step toward increase in the immunogenicity of MSCs. Immunoproteasome is large proteolytic machinery, it is abundantly expressed in immune cells, such as antigen-presenting cells^57,58^. The immunoproteasome has been involved in the pathogenesis of several inflammatory diseases such as autoimmune disease^47,57^. Therefore, blocking immunoproteasome is believed to be a clinically relevant strategy to explore treatment for inflammatory diseases. Immunoproteasome has also been reported to play a role in skeletal muscle differentiation^59,60^. However, the role of immunoproteasome in HLA-DRα activation and immunogenicity of MSCs has not been reported yet. HLA-DRα molecule is assembled in endoplasmic reticulum, where it associates with invariant chain- Ii/CD74, that masks the antigen-binding groove, and antigenic peptides cannot bind to HLA-DRα^51,52^. On the other hand, cleavage of HLA-DRα from Ii/CD74, and its association with HLA-DM facilitates activation of HLA-DRα and its binding to antigenic peptides that promotes antigen processing. In the current study, we found that blocking immunoproteasome formation in hypoxic MSCs prevented association between HLA-DRα and HLA-DM, it rather promoted binding between HLA-DRα and Ii/CD74^51^. These data confirm that hypoxia-induced formation of immunoproteasome is responsible for maturation and activation of HLA-DRα in MSCs. In this regard, previous studies have reported that immunoproteasome mediates antigen presentation role of MHC-I in immune cells by preparing and loading antigenic peptides on MHC-I to alert host CD8^+^ T cells^46^. However, current study is the first to demonstrate the role of immunoproteasome in HLA-DRα activation. Furthermore, findings in the current study also confirm that blocking immunoproteasome formation in hypoxic MSCs preserves their immunoprivilege. Therefore, these observations provide unique insight into the mechanisms responsible for hypoxia or ischemia-induced increase in the immunogenicity of allogeneic human MSCs. Bone marrow-derived allogeneic MSCs are in clinical trials for treating wide range of inflammatory and degenerative diseases^20,61^. The outcome of initial clinical trials reported beneficial effects of transplanted MSCs^19,20,56^. However, a major limitation acknowledged by experts in the field is poor survival of transplanted MSCs in the recipient. In fact, it is now established that even though MSCs are immunoprivileged under in vitro conditions, after transplantation in the host tissue MSCs become immunogenic and are rejected by host immune system^29,30,62^. Therefore, the outcome of current study may provide molecular targets to plan interventions to prevent rejection of transplanted MSCs in the hypoxic or ischemic environment.

## Materials and methods

### Human mesenchymal stem cells

Bone marrow-derived-human MSCs were purchased from Lonza (PT 2501 CA10064-080). All the human MSCs based studies were approved by the University of Manitoba’s Research Ethics Board.

### Experimental treatments

Hypoxia treatment was employed for 24 h, the culture plates were placed in hypoxia chamber (oxygen level regulated at 0.2–0.4%) in the incubator (Biospherix hypoxia chamber). To block 26S proteasome, MSCs were treated with a specific inhibitor MG132 (2 µM and 5 µM) for 24 h. Pharmacological inhibition of immunoproteasome in MSCs was done by treating the cells with Onx0914 (0.5 µM and 1 µM) for 4 h.

### Immunoproteasome inhibition by siRNA

siRNA against LMP2 (siGENOME human PSMB9, Cat # M-006023-02-0005, Dharmacon) and LMP4 (siGENOME human PSMB8, Cat # M-006022-01-0005, Dharmacon) genes were employed to block immunoproteasome in hMSCs. Non-targeting siRNA (Cat # D-001206-13-05, Dharmacon) was used as a control. We used Lipofectamine®RNiMax (Invitrogen) to transfect 2 × 10^5^ hMSCs/well with 50 pmoles of siRNA following manufacturer’s instructions.

### Western blot

The protein levels for HLA-DRα, PSMD11, PSMD4, β1, β2, β5, PSMA1, PSMA4, PA28α, LMP2, LMP7, and MECL-1 were measured by Western blot using specific antibodies as described in our previous studies^10,31^. Briefly, total protein levels were measured by Bradford method and 35 μg of protein was loaded onto SDS-PAGE. After separation with SDS-PAGE, proteins were transferred to PVDF membrane and incubated with appropriate primary and secondary antibodies. The membranes were developed using X-ray film, and bands were quantified using Quantity One software for densitometry.

### Immunoprecipitation

To study protein-protein interactions, immunoprecipitation was performed using manufacturers’ guidelines (Santa Cruz Biotechnology). Briefly, cell lysates were prepared from different treatment groups and precleared using appropriate preclearing matrix. To form IP antibody-IP matrix complex, 20 µl of suspended (25% v/v) IP matrix, 1–5 µg of IP antibody in 500 µl of PBS were incubated overnight at 4 °C. Total cellular protein (300 µg) was transferred to the pelleted matrix and incubated overnight at 4 °C. The protein samples were then analyzed using SDS-PAGE as described for the western blotting procedure and were probed with primary and secondary antibodies. The membranes were developed using X-ray films, and quantification of bands was performed using Quantity One software.

### Immunocytochemistry

Immunohistochemistry was performed to measure expression of PSMD11, PSMD4, β1, β2, β5, PA28α, LMP2, LMP7, and MECL-1 in MSCs as described in our previous studies^11,31^. The cells were seeded onto sterile coverslips, and allowed to expand until 60% confluency. MSCs were fixed with 4% PFA and permeabilized using 0.2% Triton X in PBS at room temperature. The cells were then stained with primary and secondary antibodies as well as phalloidin (for F-actin). Nuclei were stained with DAPI. Finally MSCs were imaged using Cytation 5 system (BioTek Instruments).

### Measurement of 26S proteasome activity

26S proteasome activity was measured by determining the proteolytic activity of 20S subunit using a kit purchased from Cayman Chemicals (10008041). The fluorescent substrate (SUC-LLVY-AMC) in the kit was used to determine the activity and fluorescent intensity of each well was read at 350 nm (excitation) and 480 nm (emission).

### Immunoproteasome activity assay

To determine immunoproteasome activity, we measured proteolytic activities of β1i/LMP2 and β5i/LMP7 subunits, using fluorescent substrates- S310 (Ac-PAL-AMC) and S-320 (Ac-ANW-AMC) respectively. The fluorescence intensity of each well was read at 345 nm (excitation) and 445 nm (emission).

### Leukocyte mediated cytotoxicity

To measure leukocyte mediated cytotoxicity in MSCs, commercially available human leukocytes were purchased from Stem Cell Technologies (Cat# 70025) and were co-cultured with allogeneic hMSCs as described in our previous studies^10,31^. Briefly, hMSCs were exposed to hypoxia (0.2%–0.4% O_2_ for 24 h), and then co-cultured with leukocytes at a ratio of 1:10 (hMSCs: leukocytes). After 72 h of co-culture, leukocyte-mediated cytotoxicity in MSCs was determined by measuring the lactate dehydrogenase (LDH) which was released from the damaged MSCs (LDH Cytotoxicity Detection Kit; Clontech). The cytotoxicity in hMSCs was also measured by using RealTime-Glo™ Annexin V Apoptosis live assay (Promega, Cat# JA101, Lot # 0000390132) following the manufacturer’s guidelines.

### Leukocyte proliferation assay

Leukocyte proliferation after 72 h of co-culture with MSCs was measured using the commercial kit (Biovision, Catalog #K301). Briefly, MSCs were seeded in 96 well plate (5 × 10^4^ cells/well) and incubated for 24 h. Then cells in different groups were co-cultured with allogenic human leukocytes (Stem Cell Technologies, Cat# 70025) at a ratio of 1:10. Leukocytes were pre-activated in the presence of 10 μg/ml phytohemagglutinin (PHA) for 24 hr. After 72 hr of co-culture, leukocytes suspended in the media were transferred to another 96 well plate, followed by the addition of 10 µl of WST1 solution to each well. After incubation for 2 h, the absorbance values were taken at 450 nm using Cytation 5 system (BioTek Instruments).

### Regulatory T cell measurement assay

The number of CD4^+^CD25^+^FOXP3^+^ regulatory T cells (Tregs) were counted using flow cytometry (BD Accuri^TM^ C6) in the total leukocyte population after 72 h of co-culture with allogeneic MSCs as described in our previous studies^11,31^. Leukocytes were washed and subsequently stained with following monoclonal antibodies: FITC anti-rat CD4 (W3/25, BioLegend 201505), PE anti-rat CD25 (OX-39, BioLegend 202105), and Alexa Flour® 647 anti-mouse/rat/human FOXP3 (150D, BioLegend 320014). Appropriate isotype controls and a viability stain (BD Horizon^TM^. Fixable Viability Stain 620, BD Biosciences 564996) were used. Lymphocytes were identified by their forward and side-scatter profiles and subsequently were gated on the CD4^+^ T-cells from which the CD25^+^FOXP3^+^ subpopulation was identified.

### Statistical analysis

Data were reported as mean ± SD. Comparison of data between multiple groups was performed using one-way analysis of variance (ANOVA) followed by Tukey’s post-hoc multiple comparison test, and analysis between two groups was made using Student’s *t*-test (two-tailed). Statistical significance is determined as *p* < 0.05.

## Supplementary information

Supplementery Figure Legends

Supplementary Figure 1

Supplementary Figure 2

Supplementary Figure 3
