# Supplementary material for: Hypoxia-induced shift in the phenotype of proteasome from 26S toward immunoproteasome triggers loss of immunoprivilege of mesenchymal stem cells
Source: Cell Death Dis. 2020 Jun 4;11(6):419. doi: 10.1038/s41419-020-2634-6 (PMC7272449; doi:10.1038/s41419-020-2634-6)
Supplement: Supplementary file 1 — Supplementery Figure Legends [file 41419_2020_2634_MOESM1_ESM.docx]

**Supplementary Figure Legends:**

**Supplementary Figure 1: siRNA mediated knock down of LMP2 and LMP7 in hMSCs inhibits immunoproteasome activity:** Human bone marrow-derived MSCs were transfected with siRNAs against LMP2 and LMP7 for 48 hr followed by hypoxia treatment for 24 hr. (a) Immunoproteasome activity increased in hMSCs after exposure to hypoxia compared to normoxic cells, and knocking down LMP2 and LMP7 prevented hypoxia induced increase in immunoproteasome activity. (b) HLA-DRα protein levels (Western blot) increased significantly in hypoxic hMSCs compared to normoxic cells. siRNA knock down of immunoproteasome prevented hypoxia increase in HLA-DRα. Decreased expression of LMP7 after siRNA treatment in hypoxic hMSCs confirms immunoproteasome knock down. n=4. *p<0.05 compared to normoxic hMSCs, # p<0.05 compared to hypoxic hMSCs. Each experiment was repeated 3-4 times.

**Supplementary Figure 2: Hypoxia induced formation of immunoproteasome leads to upregulation of HLA-DRα in hMSCs.** Human bone marrow-derived MSCs were treated with hypoxia for 24 hr. (a) Western blot analysis revealed a significant increase in HLA-DRα protein levels in hypoxic hMSCs compared normoxic cells, treatment with immunoproteasome inhibitor (ONX0914) downregulated hypoxia induced increase in HLA-DRα levels. n=3. (b) HLA-DRα expression by immunostaining increased in hypoxic hMSCs versus normoxic cells, treatment with ONX0914 downregulated hypoxia induced increase in HLA-DRα expression. n=3. *p<0.05 compared to normoxic MSCs, # p<0.05 compared to hypoxic MSCs. Each experiment was repeated 3-4 times.

**Supplementary Figure 3: siRNA mediated knock down of immunoproteasome preserved immunoprivilege of hypoxic hMSCs.** To investigate the immunogenicity of MSCs, normoxic and hypoxic hMSCs were co-cultured with allogeneic leukocytes at a ratio 1:10 for 72 hr. (a) Leukocyte mediated cytotoxicity in MSCs was measured using RealTime-Glo™ Annexin V Apoptosis live assay. The cytotoxicity level increased in hypoxic hMSCs versus normoxic cells, which was recovered by either ONX0914 treatment (0.5μM for 4hr) or siRNA mediated inhibition of immunoproteasome. n=6. (b) The effect of MSCs on leukocyte proliferation was measured using WST1 proliferation assay kit. After 72 h of co-culture, normoxic MSCs were able to decrease leukocyte proliferation compared to control (PHA treated leukocytes). However, hypoxia treated MSCs had no effect on leukocyte proliferation, knocking down immunoproteasome using siRNA in hypoxic MSCs significantly decreased leukocyte proliferation. n=6. @p<0.05 compared to PHA group; #p<0.05 compared to hypoxic MSCs, each experiment was repeated 3-4 times.
