# Supplementary figures and images for: Hypoxia-induced shift in the phenotype of proteasome from 26S toward immunoproteasome triggers loss of immunoprivilege of mesenchymal stem cells
Source: Cell Death Dis. 2020 Jun 4;11(6):419. doi: 10.1038/s41419-020-2634-6 (PMC7272449; doi:10.1038/s41419-020-2634-6)

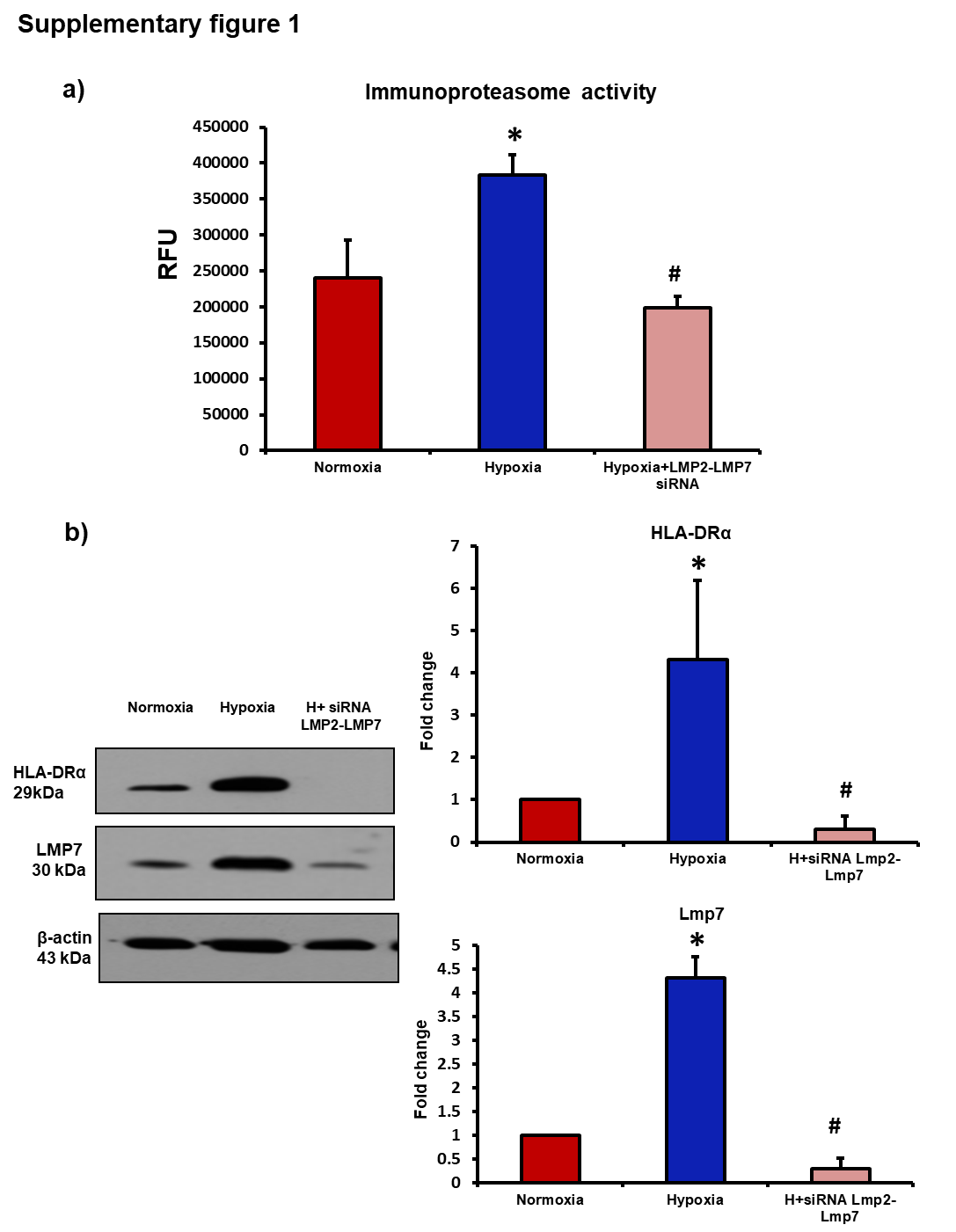

Supplement: Supplementary file 2 — Supplementary Figure 1 [file 41419_2020_2634_MOESM2_ESM.tif]

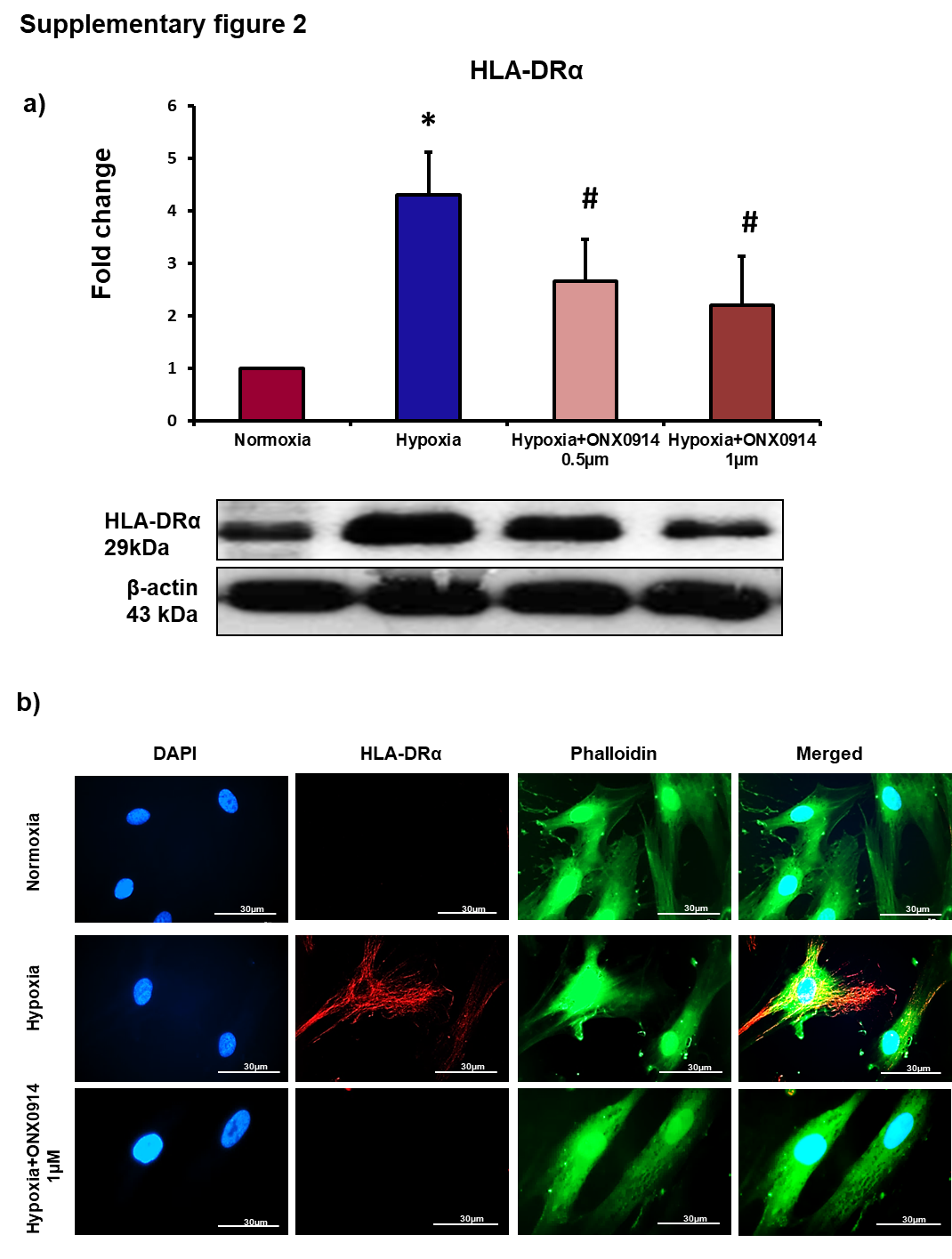

Supplement: Supplementary file 3 — Supplementary Figure 2 [file 41419_2020_2634_MOESM3_ESM.tif]

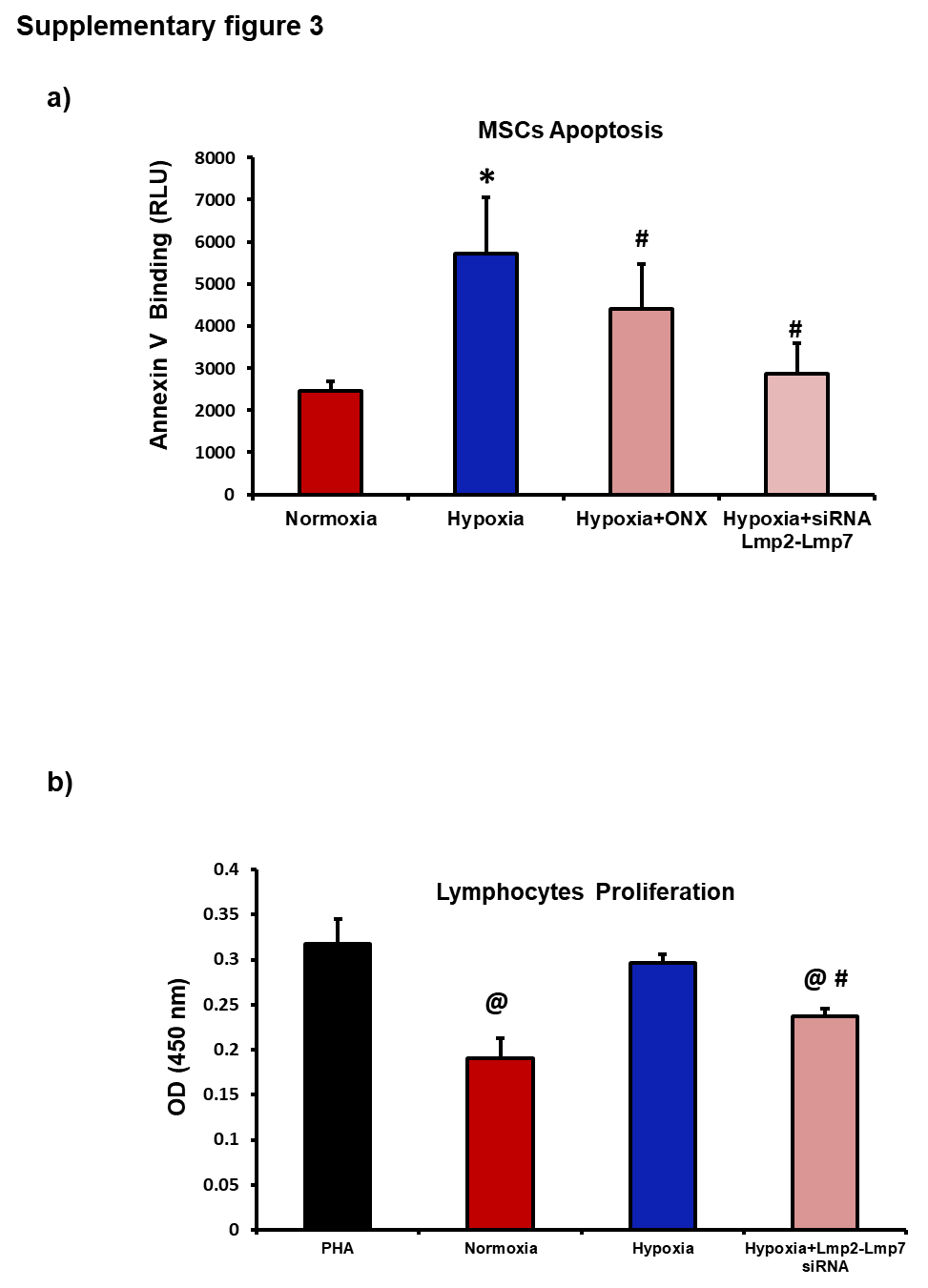

Supplement: Supplementary file 4 — Supplementary Figure 3 [file 41419_2020_2634_MOESM4_ESM.tif]
